# Supplementary material for: Complexome profiling of the Chlamydomonas psb28 mutant reveals TEF5 as an early PSII assembly factor
Source: Plant Cell. 2025 Mar 17;37(6):koaf055. doi: 10.1093/plcell/koaf055 (PMC12164590; doi:10.1093/plcell/koaf055)
Supplement: koaf055_Supplementary_Data [file koaf055_supplementary_data.zip › psb28_interactive.zip!/plots/intensity/qualityPlots/peptides/OTA1_Cre11.g474800.t1.2.html]

### Cre11.g474800.t1.2

Trivial name: OTA1  
SeqCoverage [median]: 24.70  
Mass: 48.29 kDa  
  
 

| Sequence | MissedCleavage | Mass [Da] | wtIntensity | muIntensity | Score |
| --- | --- | --- | --- | --- | --- |
| ALSGGMYPVSAALAR | 0 | 1462.76 | 665.27 | 1472.32 | 106.67 |
| DNGLLAKPTQK | 1 | 1183.66 | 0.00 | 326.09 | 44.54 |
| FGAHNYAPIPVVLDR | 0 | 1667.88 | 0.00 | 930.02 | 46.96 |
| GQHGSTYGGNPLAAK | 0 | 1456.71 | 328.92 | 1856.00 | 50.16 |
| IVEALTSQAGK | 0 | 1115.62 | 1812.92 | 3613.68 | 109.66 |
| LAAIPSK | 0 | 698.43 | 862.07 | 2900.52 | 88.73 |
| VAMAALQVLQDER | 0 | 1442.76 | 1723.24 | 8507.32 | 162.36 |
| VLPMNTGVEGGETAIK | 0 | 1614.83 | 747.09 | 3645.15 | 88.27 |
|  |  |  | 6139.5 | 23251.1 |  |
